# Frequency and neuropathology of *HTT* repeat expansions in FTD/ALS: co-existence rather than causation

**DOI:** 10.1007/s00415-024-12822-2

**Published:** 2024-12-12

**Authors:** Milan Zimmermann, David Mengel, Katrin Raupach, Tobias Haack, Manuela Neumann, Matthis Synofzik

**Affiliations:** 1 Department of Neurodegenerative Diseases, Hertie-Institute for Clinical Brain Research and Center of Neurology, Tuebingen University Hospital, Hoppe-Seyler-Str. 3, 72076 Tuebingen, Germany; 2 German Center for Neurodegenerative Diseases (DZNE), Otfried-Müller-Str. 23, 72076 Tuebingen, Germany; 3 Research Division Translational Genomics of Neurodegenerative Diseases, Hertie-Institute for Clinical Brain Research, Hoppe-Seyler-Str. 3, 72076 Tuebingen, Germany; 4 Division of Molecular Genetics, Tuebingen University Hospital, Calwerstr. 7, 72076 Tuebingen, Germany; 5 Department of Neuropathology, Tuebingen University Hospital, Calwerstr. 3, 72076 Tuebingen, Germany

**Keywords:** Huntington, ALS, Amyotrophic lateral sclerosis, Frontotemporal dementia, HTT, MAPT

## Abstract

**Introduction:**

While ≥ 40 CAG repeat expansions in *HTT* present a well-established cause of Huntington’s disease (HD), an enrichment of *HTT* repeat expansions was recently reported also in patients with amyotrophic lateral sclerosis (ALS) and frontotemporal dementia (FTD), including FTD/ALS patients with additional HD neuropathology. This raises the question whether the phenotypic spectrum of *HTT* expansions can be extended to ALS and FTD, and whether *HTT* should be considered as a new causative gene of FTD/ALS. If *HTT* repeat expansions were indeed systematically related to FTD/ALS, one would expect an increased frequency of *HTT* carriers in FTD/ALS, who can clinically/neuropathologically not be explained better than by the presence of the *HTT* repeat expansions.

**Methods:**

Screening of *HTT* repeat expansions in 249 consecutive patients with ALS or FTD by short-read genome sequencing took place. The post-mortem neuropathological examination was performed in the identified *HTT* repeat expansion carrier.

**Results:**

One *HTT* repeat expansion [40/22 repeats (± 1)] was identified in an ALS patient, giving a frequency of 0.4% (1/249) (frequency in the general population: 0.03–0.18%). This patient showed a classic ALS phenotype, but no clinical or imaging signs of HD. Post-mortem brain examination revealed—in addition to ALS-typical degeneration of upper and lower motor neurons with TDP-43 inclusions—HD-typical polyQ-aggregates in gyrus cinguli, striatum and frontal lobe, yet without evidence of striatal degeneration.

**Conclusions:**

Our study does not support the notion of an increased frequency of *HTT* repeat expansions in FTD/ALS. Moreover, the phenotype of the *HTT* carrier identified can be better explained by two co-existent, but independent diseases: (i) ALS and (ii) presymptomatic HD, which—given the low repeat number—is likely to become manifest only later in life. These findings corroborate the concept that *HTT* repeat expansions are likely co-existent/coincidental, but not causative in FTD/ALS.

**Supplementary Information:**

The online version contains supplementary material available at 10.1007/s00415-024-12822-2.

## Introduction

While ≥ 40 CAG repeat expansions in *HTT* present a well-established cause of Huntington`s disease (HD), an enrichment of *HTT* repeat expansions was recently reported also in patients with amyotrophic lateral sclerosis (ALS) or frontotemporal dementia (FTD), including FTD/ALS patients with additional HD neuropathology [1]. Specifically, Dewan and colleagues found: (i) a frequency of 0.12% (3/2442) and 0.14% (5/3674) *HTT*-carriers, respectively, in FTD/ALS cohorts compared to 0.03% (10/31372) in the general population; and (ii) both FTD/ALS-typical TDP-43-pathology and HD-typical polyQ-inclusions without evidence of striatal degeneration in autopsy of two *HTT*-carriers with an ALS-phenotype [2]. These findings raise the question whether the phenotypic spectrum of *HTT* expansions can be extended to ALS and FTD, and whether *HTT* should be considered as a new causative gene of FTD/ALS.

Given the recent controversy on this notion [3, 4] and the need for validation by independent screening studies and post-mortem studies (as emphasized by the authors themselves, [1, 5]), we here investigated the following two-fold hypothesis: if *HTT* repeat expansions were indeed systematically related to FTD/ALS, one would expect (1.) an increased frequency of *HTT* carriers in FTD/ALS, (2.) who can clinically/neuropathologically not be explained better than by the presence of the *HTT* repeat expansions. This hypothesis was tested by a large screening of *HTT* repeat expansions by short-read genome sequencing (SR-GS) of 249 patients with ALS or FTD, combined with post-mortem neuropathological examination in the *HTT* carrier identified by this large screening.

## Methods

### Genome-based HTT expansion screening of a consecutive FTD/ALS series and in-depth phenotyping

A consecutive series of 249 subjects with ALS (N = 188), FTD (N = 52) or FTD/ALS (N = 9)—each diagnosed to standard criteria (ALS: [6–8]; FTD: [9, 10]; FTD/ALS: [11])—was recruited by the FTD/ALS outpatient clinics of the Center of Neurology, Tuebingen, between 2019 and 2022 and investigated by short-read genome sequencing (SR-GS). The sequencing libraries were generated using the Illumina DNA PCR-Free protocol and sequenced on an Illumina NovaSeq 6000 sequencer with a target depth of 38x. The sequencing reads were mapped to the GRCh38 reference genome using BWA-mem2 v.2.2.1 (https://github.com/bwa-mem2/bwa-mem2) and repeat expansions were detected with ExpansionHunter v5.0.0 (https://github.com/Illumina/ExpansionHunter). In-depth phenotyping was performed in the identified patient with a pathological CAG *HTT* expansion by clinical, imaging, electrophysiological and fluid biomarker studies, followed by autopsy and post-mortem neuropathology examination. All subjects provided written informed consent before participation and publication according to the Declaration of Helsinki.

### Neuropathological examination

The identified patient with a *HTT* repeat expansion, who died at age 63 years, underwent autopsy of brain and spinal cord, performed at the Brain Bank Unit Tuebingen of the DZNE Brain Bank. Neuropathological evaluation was performed on formalin-fixed paraffin embedded tissue sections from 20 standardized neuroanatomical regions (including various neocortical regions, basal ganglia, thalamus, amygdala, hippocampus, brain stem, cerebellum and spinal cord) following guidelines for the assessment and diagnosis of neurodegenerative diseases including hematoxylin and eosin staining and immunohistochemistry with antibodies against phosphorylated TDP-43 (clone 1D3, [12]), phosphorylated tau (clone AT8, Thermo Fisher), α-synuclein (clone 4D6, Origene), beta-amyloid (clone 4G8, Covance), polyQ (clone 1C2, Millipore), p62 (BD Transduction Laboratories) and GFAP (clone GA5, Diagnostic BioSystems) using the Ventana BenchMark XT automated staining system with the Optiview DAB detection kit (Ventana). The 1C2 antibody against polyQ is widely used in neuropathological evaluation of postmortem HD brains against expanded polyglutamine tracts [13, 14].

## Results

### Frequency of HTT repeat expansions in FTD/ALS

*HTT* repeat expansion screening by SR-GS in 249 FTD/ALS patients identified one ALS patient with a pathogenic *HTT* repeat expansion (40/22 CAG repeats (± 1) (repeat size confirmed by fragment length analysis)), giving a frequency of 0.4% (1/249) (*HTT* repeat expansion allele frequency in the literature: 0.03 [2]—0.18% [15]) (for cohort characteristics in terms of family history and further genetic findings, see Supplement 1). 18 patients (7.2%) had a predicted intermediate expansion within the range 27–35 CAG repeats; 1 patient a pathogenic repeat expansion with reduced penetrance (37 CAG repeats).

No other second mutation was identified in the ALS patient with a pathogenic *HTT* repeat expansion which might have explained either his clinical ALS phenotype or the late-onset dementia syndrome in his ancestors (see below); except a variant in *microtubule associated protein tau (MAPT)*, NM_001123066.4: c.509del, p.(Pro170LeufsTer24), GRCh38(chr17):g.45983312del. This was formally categorized as variant of uncertain significance (VUS), but was unlikely to have a pathogenic impact as it was located only deep-intronic in the main brain expressed MAPT transcripts (ENST00000351559.10, ENST00000446361.7, ENST00000535772.6). Furthermore, in those weakly brain expressed transcripts, where it was located exonic (ENST00000262410.10, ENST00000344290.10, ENST00000415613.6, ENST00000571987.5), it predicts a premature stop – yet loss-of-function does not present a mutational mechanism known to lead to disease in *MAPT*. Two wildtype *C9orf72* alleles were predicted by ExpansionHunter, and a *C9orf72* repeat expansion was additionally also ruled out by PCR-based fragment length analysis (for further details on the sequencing statistics and coverage of genes of interest, see Supplement 2).

### In-depth phenotyping

**Fig. 1:**
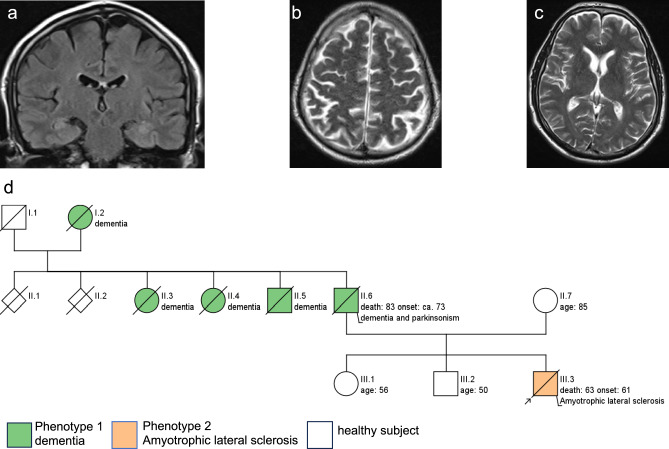
Cerebral magnetic resonance imaging (cMRI) in the index patient. cMRI at age 61 years revealed no evidence of atrophy in the frontal lobe, insula or caudate nucleus (**a**: frontal T2 FLAIR image showing intact insula; **b**: axial T2-weighted image demonstrating absence of frontal atrophy; **c** axial T2-weighted image indicating no caudate nucleus atrophy). Pedigree of the index patient. Family history of the index patient (marked by an arrow) is positive for Parkinson`s disease and dementia (**d**). His father was diagnosed with a “Parkinsonian syndrome” at about age of 60 years, developed symptoms of a dementia with 73 years comprising language decline and changes in behaviour, and died at age 83 years. Two paternal aunts and one paternal uncle out of in total six siblings as well as their mother were also diagnosed with a late-onset neurodegenerative dementia syndrome. None of the relatives were suffering from ALS

The male patient showed a classic ALS phenotype, with disease onset at age 61 years with progressive dysarthria, dysphagia, paralysis and death after 2 years due to global respiratory insufficiency. The neurological examination revealed an involvement of both upper and lower motor neuron, without any clinical signs or changes of behaviour characteristic of HD even on repeated investigations by independent movement disorders neurologists. Furthermore, the patient scored only 8 points on the Unified Huntington's Disease Rating Scale (UHDRS [16]), likely reflecting the effects of ALS rather than (even incipient) HD, as they included: gait disturbances from paresis, severe dysarthria, and difficulties with tandem walking. There were no specific clinical signs of HD, including no abnormalities of tongue protrusion, chorea or dystonia. CSF NfL levels were substantially increased to 4090 pg/mL (cut-off < 916 pg/mL). Cerebral and spinal MRI ruled out competing diagnoses like ischemic lesions, tumors and spinal stenosis. No regional atrophies including frontal lobe, insula, striatum and caudate nucleus were found (see Fig. 1a, b, c). Overall Huntington's Disease Integrated Staging System (HD-ISS [17]) disease stage was 0. Family history was positive for late-onset (> 60 years) dementia syndromes in several generations, including reported behavioural changes, progressive speech decline and parkinsonism in the index patient´s father (see Fig. 1d), indicating an autosomal-dominant family history for a, partly complex, late-onset neurodegenerative dementia syndrome.

### Neuropathology

**Fig. 2:**
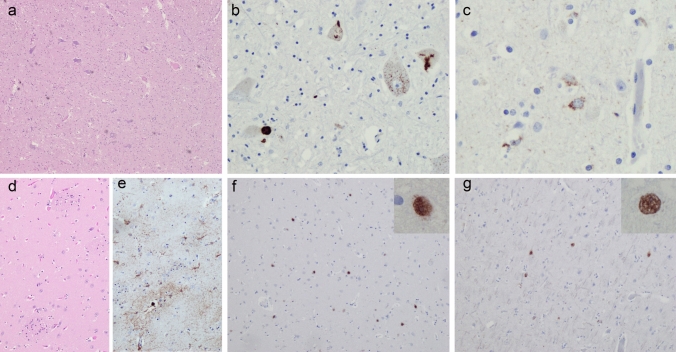
Neuropathology. Typical ALS pathology with (**a**) loss of motor neurons in the anterior horn of the spinal cord (H&E) and TDP-43-immunoreactive neuronal cytoplasmic inclusions in the spinal cord (**b**) and precentral gyrus (**c**). (**d**) The caudate nucleus revealed no signs of neurodegeneration by H&E. However, mild astrogliosis was detected by GFAP immunohistochemistry (**e**). PolyQ-immunoreactive neuronal intranuclear inclusions were present in the striatum (**f**) and frontal cortex (**g**)

Macroscopically, the spinal cord revealed atrophic anterior roots, otherwise the CNS was unremarkable. The histological analysis showed mild to moderate loss of Betz’s cells in the precentral gyrus, as well as moderate loss of motor neurons in the hypoglossal nucleus and anterior horns of the spinal cord (Fig. 2a). TDP-43 immunoreactive inclusions, characteristic for ALS (neuronal cytoplasmic inclusions and oligodendroglial inclusions), were present in the spinal cord, brain stem and precentral gyrus (Fig. 2b, c).

No obvious cell loss and gliosis were seen in H&E stains in HD-characteristic brain regions such as the caudate nucleus (Fig. 2d) and putamen. However, GFAP-immunohistochemistry revealed mild to moderate gliosis in the head of the caudate nucleus (Fig. 2e), in line with Vonsattel grade 1 [18]. Moderate numbers of anti-polyQ labelled neuronal nuclei as well as more compact intranuclear inclusions were found in the striatum (Fig. 2f), frontal cortex (Fig. 2g) and gyrus cinguli, as characteristic for HD.

As an additional minor comorbid finding, mild Alzheimer’s Disease associated neuropathological changes (ABC score A2, B1, C1) were present.

## Discussion

Our work tested the recently reported hypothesis that the phenotypic spectrum of *HTT* expansions might be extended to ALS and FTD, and that, correspondingly, *HTT* might be considered as a new causative gene, or at least genetic risk factor, of FTD/ALS [1]. Combining a large-scale genetic screening plus in-depth phenotyping and post-mortem neuropathology investigations, we did not find an increased frequency of *HTT* repeat expansions in 249 WGS datasets (1/249 = 0.4%, compared to the *HTT* repeat expansion allele frequency of 0.03 [2]—0.18% [15] in the general population). Moreover, the phenotype and neuropathology of the only *HTT* repeat expansion carrier identified by this large screening was better explained by two independent diseases: (i) ALS and (ii) as of yet still presymptomatic HD stage, which, given the low repeat number, is likely to become manifest only later in life. Given that polyQ-inclusion pathology was still mild, and atrophies absent, it is more parsimonious to assume that this very mild HD neuropathology is indicative of a presymptomatic HD stage, rather than the putative cause of the full-blown, fatal ALS disease.

These results question the presumed causality of *HTT* repeat expansions in FTD/ALS. This notion had been especially based in a prior study on two findings: (i) a frequency of 0.12% (3/2442) and 0.14% (5/3674) *HTT*-carriers, respectively, in FTD/ALS cohorts compared to 0.03% (10/31372) in the general population; and (ii) both FTD/ALS-typical TDP-43-pathology and polyQ-inclusions without evidence of striatal degeneration in autopsy of two *HTT*-carriers with an ALS-phenotype [1]. These findings have already been criticized on conceptual grounds [3]: (i) The clinical phenotype with cognitive symptoms and language disturbance described in five out of eight presumable “FTD patients” of this series might be compatible with HD (especially in one patient with apathy and irritability) rather than with FTD, rendering it likely that the observed *HTT* repeat expansions simply caused HD, rather than FTD. (ii) In the three identified ALS patients with *HTT* repeat expansions, the CAG repeat expansions size was near the threshold of 40 repeats, rendering it possible that also in these three instances, like probably the case in the patient reported here, these respective patients were carrying two independent conditions: a manifest ALS disease, and an, as of yet presymptomatic, Huntington’s disease. This would also present the most parsimonious explanation for the neuropathology investigations performed in two of the three subjects: the presence of polyQ and p62 staining, in the absence of striatal degeneration, is best indicative of a presymptomatic HD stage [19]. Given the well-established correlation between repeat length and severity of clinical symptoms, age at onset and extent of striatal degeneration [20] as well as the small expansion of the repeat size, HD would only become manifest later in life in these subjects, allowing other concomitant diseases, such as ALS, to manifest earlier in life. Supporting this notion, in the 63-year-old subject reported here with a small expansion of the *HTT* repeat, we also found typical signs of full-blown ALS pathology with atrophy of first and second motor neuron, accompanied by TDP-43-positive inclusions; but with only mild levels of polyQ- aggregates without striatal atrophy, in sum indicative of concomitant HD, which was, in contrast to the ALS disease, yet a still very early stage. HD would likely have become manifest later in life, possibly with a complex late-onset neurodegenerative dementia syndrome as in the patient’s ancestral generations.

This notion proposed here would also provide the most parsimonious explanation for other recent findings. Hickman et al. found both HD and ALS neuropathology in 6 out of 751 brains from the New York Brain Bank. While exceeding the prevalence of ALS in the USA (0.8% vs. 0.0052%), all of these patients had *HTT* repeat expansions in the range from 41 to 44 CAG repeats [21], and detailed clinical information was available only for 2/6 subjects. In at least 1 of the 2 cases, where clinical information was available (Case #1; 41 CAG repeats; ALS disease, but only HD neuropathology grade 1), the *HTT* repeat expansion is likewise most parsimoniously explained as a coincidental finding of a not yet-manifest HD disease, coexistent, but not causative, to the ALS disease. Correspondingly, like our study, another study could also not confirm an increased frequency of pathogenic *HTT* repeat expansions in ALS, observing only 2 patients with intermediate, but no patient with pathogenic *HTT* repeat expansions in a screening cohort of 608 ALS patients [22]. However, our study is limited by the fact that we identified only a single ALS patient with a *HTT* repeat expansion, allowing no robust statistical comparison on the *HTT* repeat carrier frequency in our ALS cohort vs general population. To test this further, future studies of additional larger existing FTD/ALS cohorts vs general population data—ideally from the same ethnic origin and sequencing technique—are warranted.

In summary, our findings suggest a parsimonious notion to explain our and others’ observations of *HTT* repeat expansions in FTD/ALS patients. Specifically, they suggest that there is yet no sufficient evidence for extending the phenotypic spectrum of *HTT* mutations beyond HD to include also ALS and FTD phenotypes. A more likely explanation is that, in some patients, *HTT* repeat expansions are simply a coincidental finding, at a yet presymptomatic HD stage, coexistent, but not causative, to an ALS/ FTD disease.

## Supplementary Information

Below is the link to the electronic supplementary material.

Supplementary file1 (DOCX 15 KB)

## Data Availability

The pseudonymized data of this study are available from the corresponding author upon reasonable request.
