# Supplementary material for: Frequency and neuropathology of HTT repeat expansions in FTD/ALS: co-existence rather than causation
Source: J Neurol. 2024 Dec 12;272(1):58. doi: 10.1007/s00415-024-12822-2 (PMC11638390; doi:10.1007/s00415-024-12822-2)
Supplement: Supplementary file 1 — Supplementary file1 (DOCX 15 KB) [file 415_2024_12822_MOESM1_ESM.docx]

**Supplementary Material**

**Supplement 1: Cohort characteristics: frequency of familial FTD/ALS and other genetic findings**

Family history data was available for 171 out of the 249 patients in the cohort. Out of 188 ALS patients, 18 had a family history of ALS (9.6%), and 4 (2.1%) had a family history of dementia. Among the 52 patients with FTD, 18 (34.6%) had a family history of dementia, including 5 patients with relatives who had late-onset dementia. Among the 9 patients with FTD/ALS overlap syndrome, 3 had a family history of dementia (33.3%) and 1 (11.1%) had a family history of ALS.

A firm molecular diagnosis (likely pathogenic / pathogenic changes) in any ALS- or ALS-spectrum gene was identified in 7 individuals of this cohort (3 *C9orf72*, 1 *OPTN*, 1 *TBK1*, 1 *ANG,* 1 *GRN*). The low/absent yield of *SOD1* and *C9orf72* repeat mutations might be explained by the fact that this cohort had already largely been screened for *SOD1* and *C9orf72* mutations by single gene analysis prior to WGS.

**Supplement 2: Genetic sequencing statistics and coverage of genes of interest**

Genes prioritized for analysis from the WGS dataset based on a broad characterization of the patients’ phenotypic features by HPO terms. [HPO: HP:0003202 (Skeletal muscle atrophy), HPO: HP:0007354 (Amyotrophic lateral sclerosis), HPO: HP:0007373 (Motor neuron atrophy)]

The target region of each gene comprised at least the consensus coding sequence (CCDS) of the genes listed below ±20 bases of flanking intronic sequence, but may also include additional exons and/or flanking bases.

*ABCA2, ACBD5, ADGRB2, AIF1L, AK9, AKR1C3, ALAD, ALS2, ANAPC7, ANG, ANXA11, APEX1, APOE, AR, ARHGAP33, ARHGEF10, ARHGEF28, ARPP21, ATL1, ATM, ATP13A2, ATP7A, ATP8B3, ATXN1, ATXN2, BICD2, C9ORF72, CACNA1A, CACNA1H, CAPZA1, CARS1, CCDC40, CCDC81, CCNF, CCS, CD33, CDH13, CFAP410, CFAP54, CHCHD10, CHCHD2, CHGB, CHMP2B, CHRM1, CHRNA4, CLEC4C, CNGA4, CNOT1, CNTF, COL19A1, CRYM, CSF1R, CX3CR1, CYLD, DAO, DCTN1, DDHD1, DENND2C, DHFR, DIAPH3, DNAH10, DNAH2, DNAH9, DNAJC13, DNAJC7, DPP6, DPYSL3, DYNC1H1, EEF1G, EHMT1, EIF4E1B, ELP3, EPHA4, ERBB4, ERLIN1, EWSR1, EZR, FBXO7, FGD6, FGGY, FIG4, FLNC, FOXA1, FOXN3, FUS, GARS1, GBE1, GJB1, GLE1, GLT8D1, GNE, GORASP1, GRB14, GRN, GRSF1, GSN, GTF2H4, GTF3C2, HDAC10, HENMT1, HEXA, HFE, HNRNPA1, HNRNPA2B1, HNRNPD, HNRNPU, HNRNPUL1, HSPB1, IFT74, IGHMBP2, ITPR2, KBTBD13, KIAA1755, KIF5A, KIFAP3, KMT2C, LBP, LIMD1, LMNA, LRRK2, LUM, LZTS3, MAPT, MATR3, MEF2C, METTL22, MFN2, MME, MORC2, MRPL40, MTHFR, MTHFSD, MYO1A, MYO3B, NEFH, NEK1, NIPA1, NLRC5, NME8, NOTCH3, NPC1, NTM, ODAD2, OGG1, OMA1, OPTN, P4HB, PARK7, PDIA3, PFN1, PINK1, PIP4K2C, PLA2G4C, PLCD1, PLEKHG5, POLG, PON1, PON2, PON3, PPARGC1A, PPARGC1B, PRKAR1B, PRPH, PSEN1, PSEN2, PSMB7, RAB25, RAPGEF2, RARS1, RAVER1, RBM12, RBM12B, RBM15, RBM15B, RBM45, RBM4B, RBMS2, RINL, RNASE4, SBF2, SERPINA10, SETX, SIGMAR1, SLC1A2, SLC1A3, SND1, SOD1, SPAST, SPG11, SPG7, SPTB, SQSTM1, SRCAP, SRPK2, SS18L1, STK36, SUSD1, SV2A, SYNE1, TAF15, TAF1L, TARDBP, TBK1, TF, THSD7B, TIA1, TNPO3, TREM2, TRPC3, TRPM2, TRPM7, TRPV4, TRRAP, TUBA4A, UBQLN2, UBQLN4, UNC13A, USH2A, VAPB, VCL, VCP, VEGFA, VRK1, WASHC5, WDR6, XRCC6, ZFYVE26, ZNF74*

**Coverage statistics**

**Average sequencing depth genome: 46.49**

Average sequencing depth (chrMT): 1736.97

**Coverage statistics for CCDS**

Total CCDS: 680907

CCDS with depth ≥20: 680215 (99.90%)

CCDS with depth <20: 692 (0.10%)

Coverage statistics for CCDS +-5

CCDS +-5 total: 722927

CCDS +-5 with depth ≥20: 722192 (99.90%)

CCDS +-5 with depth <20: 735 (0.10%)

**Completely covered genes:** *ABCA2, ACBD5, ADGRB2, AIF1L, AK9, AKR1C3, ALAD, ALS2, ANAPC7, ANG, ANXA11, APEX1, APOE, ARHGAP33, ARHGEF10,*

*ARHGEF28, ARPP21, ATL1, ATM, ATP13A2, ATP8B3, ATXN1, ATXN2, BICD2, C9ORF72, CACNA1A, CACNA1H, CAPZA1, CARS1, CCDC40, CCDC81, CCNF, CCS,*

*CD33, CDH13, CFAP410, CFAP54, CHCHD10, CHCHD2, CHGB, CHMP2B, CHRM1, CHRNA4, CLEC4C, CNGA4, CNOT1, CNTF, COL19A1, CRYM, CSF1R,*

*CX3CR1, CYLD, DAO, DCTN1, DDHD1, DENND2C, DHFR, DIAPH3, DNAH10, DNAH2, DNAH9, DNAJC13, DNAJC7, DPP6, DPYSL3, DYNC1H1, EEF1G, EHMT1,*

*EIF4E1B, ELP3, EPHA4, ERBB4, ERLIN1, EWSR1, EZR, FBXO7, FGD6, FGGY, FIG4, FLNC, FOXA1, FOXN3, FUS, GARS1, GBE1, GJB1, GLE1, GLT8D1, GNE,*

*GORASP1, GRB14, GRN, GRSF1, GSN, GTF2H4, GTF3C2, HDAC10, HENMT1, HEXA, HFE, HNRNPA1, HNRNPA2B1, HNRNPD, HNRNPU, HNRNPUL1, HSPB1,*

*IFT74, IGHMBP2, KBTBD13, KIAA1755, KIF5A, KIFAP3, KMT2C, LBP, LIMD1, LMNA, LRRK2, LUM, LZTS3, MAPT, MATR3, MEF2C, METTL22, MFN2, MME,*

*MORC2, MRPL40, MTHFR, MTHFSD, MYO1A, MYO3B, NEFH, NEK1, NIPA1, NLRC5, NME8, NOTCH3, NPC1, NTM, ODAD2, OGG1, OMA1, OPTN, P4HB,*

*PARK7, PDIA3, PFN1, PINK1, PIP4K2C, PLA2G4C, PLCD1, PLEKHG5, POLG, PON1, PON2, PON3, PPARGC1A, PPARGC1B, PRKAR1B, PRPH, PSEN1, PSEN2,*

*PSMB7, RAB25, RAPGEF2, RARS1, RAVER1, RBM12, RBM12B, RBM15, RBM15B, RBM45, RBM4B, RBMS2, RINL, RNASE4, SBF2, SERPINA10, SETX,*

*SIGMAR1, SLC1A2, SLC1A3, SND1, SOD1, SPAST, SPG11, SPG7, SPTB, SQSTM1, SRCAP, SRPK2, SS18L1, STK36, SUSD1, SV2A, SYNE1, TAF15, TAF1L, TARDBP,*

*TBK1, TF, THSD7B, TIA1, TNPO3, TREM2, TRPC3, TRPM2, TRPM7, TRPV4, TUBA4A, UBQLN2, UBQLN4, UNC13A, USH2A, VAPB, VCL, VCP, VEGFA, VRK1,*

*WASHC5, WDR6, XRCC6, ZFYVE26, ZNF74*

**Missing bases in genes not completely covered:** *AR (147bp), ATP7A (550bp), ITPR2 (8bp), TRRAP (30bp)*
